# Antibacterial Effect of Hypochlorous Acid Solution on Nasal Discharge from Patients with Chronic Rhinosinusitis

**DOI:** 10.1155/2018/8568694

**Published:** 2018-02-27

**Authors:** Shang-Heng Wu, Jen-Fu Lin, Rong-San Jiang

**Affiliations:** ^1^Department of Otolaryngology, Taichung Veterans General Hospital, Taichung, Taiwan; ^2^Department of Pathology and Laboratory Medicine, Taichung Veterans General Hospital, Taichung, Taiwan; ^3^Department of Medical Research, Taichung Veterans General Hospital, Taichung, Taiwan; ^4^School of Medicine, Chung Shan Medical University, Taichung, Taiwan; ^5^Department of Nursing, HungKuang University, Taichung, Taiwan

## Abstract

**Purpose:**

The antibacterial effect of hypochlorous acid (HOCl) solution on nasal secretion of patients with chronic rhinosinusitis (CRS) was investigated.

**Materials and Methods:**

Five swab specimens were collected from the middle meatus of CRS patients. The first one was placed directly in a Thanswab tube while all of the others were placed randomly into 4 glass tubes containing either HOCl solution, normal saline (NS), 75% alcohol, or povidone-iodine (PVPI) solution for one minute in the first part and for 5 minutes in the second part of the study before transfer to a Thanswab tube.

**Results:**

Bacteria were cultured from 27 of 50 specimens when they were put directly in a Thanswab tube and from 26 after soaking in HOCl solution, 27 in NS, 13 in 75% alcohol, and 25 in PVPI solution for one minute. In the second part of the study, bacteria were cultured from 14 of 32 specimens when they were put directly in a Thanswab tube and from 14 after soaking in HOCl solution, 13 in NS, 3 in 75% alcohol, and 11 in PVPI solution for 5 minutes.

**Conclusions:**

This study showed that HOCL solution did not exert an antibacterial effect on nasal secretion from CRS patients within 5 minutes.

## 1. Introduction

Hypochlorous acid (HOCl) is a weak acid which is produced when chlorine dissolves in water [1]. It can be generated by the electrolysis of a weak sodium chloride solution [2]. It has been demonstrated to have bactericidal effects [1]. Recently, HOCl solution has been used as nasal irrigant to treat pediatric chronic sinusitis [3]. Povidone-iodine (PVPI) solution is a potent antimicrobial solution [4]. Its advantages include broad dismicrobial spectrum, low cost, low risk for sensitization, and lack of bacterial resistance against the agent [5]. It has been used as mouthwash for many purposes [4].

Nasal irrigation is a popular treatment modality for various sinonasal diseases including upper respiratory tract infection, rhinitis, rhinosinusitis, and postoperative or postradiation care [6–10]. While nasal irrigation has been widely used, there is no consensus on the optimal irrigation solution [11–13]. Saline irrigation is mostly acceptable. Antimicrobial agents as antibiotics or antifungal agents had been added to the irrigation fluid [14–16]. Some researchers had also used additives as manuka honey, xylitol, or surfactant for improvement of the effect of nasal irrigation [17]. In the present work, the antibacterial effect of HOCL on the nasal secretion of CRS patients was investigated to clarify its role as nasal irrigant.

## 2. Materials and Methods 

This study was approved by the Ethics Committee of Taichung Veterans General Hospital. Written consent was obtained from each patient.

### 2.1. Study Population

A total of 82 CRS patients were collected from the outpatient clinic of the Department of Otolaryngology between September of 2015 and April of 2016. CRS was diagnosed when patients had a history of rhinosinusitis for more than 12 weeks and the nasal endoscopy found mucopurulent discharge in the nasal cavity. Those who took antibiotics within a week before were excluded from the study.

### 2.2. Study Design

The study was divided into two parts. In the first part, 5 swab specimens were collected from the ipsilateral middle meatus with greater disease severity, using a cotton-tipped stick. The first stick was put in a Thanswab tube containing 5 ml of Amies charcoal medium. The other 4 ones were each randomly put into 4 glass tubes containing 2 ml of HOCl solution, normal saline (NS), 75% alcohol, or PVPI solution. The HOCl solution was an aliquot of a bottle of NeutroPhase® Skin and Wound Cleanser (Novabay Pharmaceuticals, Inc., Emeryville, CA) which contained 0.01% HOCl solution, and the PVPI solution was an aliquot from a bottle of Betadine® Mouthwash and Gargle (Mundipharma Pharmaceuticals, Ltd., Cyprus) which contained 1% PVPI solution. All sticks were placed in glass tubes each containing one of the aforementioned solutions for one minute. Then the sticks were transferred to Thanswab tubes. All 5 Thanswab tubes were carried to the clinical microbiology department immediately. In the second part of the study, the experimental conditions were the same as in the first part of the study except that the duration was five minutes rather than one minute; that is, the sticks were placed in a solution for 5 minutes before being transferred to a Thanswab tube.

### 2.3. Bacterial Culture

In the microbiology laboratory, specimens in the Thanswab tubes were brushed on plates containing 5% sheep blood, eosin methylene blue, and chocolate agar, and the plates were placed in a 5% CO_2_ incubator at 35°C for 2 and 4 days. Specimens in the Thanswab tubes were also brushed on brucella anaerobic blood agar plates, and the plates were incubated in the Form anaerobic system for 2 and 4 days. A thioglycolate broth tube was used for enrichment of anaerobes. It was incubated at 35°C for 2 more days. All isolates were routinely identified for aerobic and anaerobic bacteria.

### 2.4. Statistical Analysis

The rates of bacterial growth of 5 groups of specimens were compared using ANOVA test. Culture results of first and second parts of the study were compared using Pearson's Chi-square test. It was considered statistically significant when *p* values < 0.05. A SPSS version 17.0 (SPSS Inc., Chicago, IL, USA) was used to perform all analyses.

## 3. Results

### 3.1. Culture Rates after Management for One Minute

**Figure 1:**
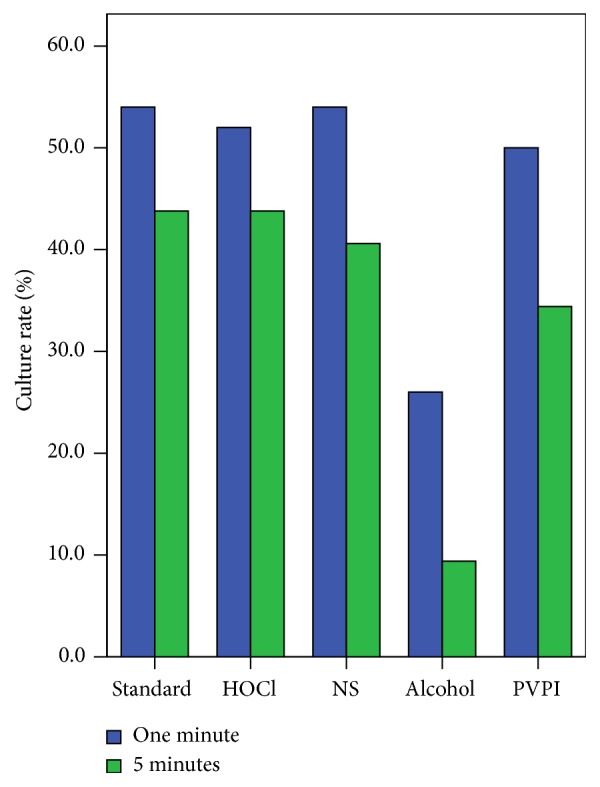
Culture rates after contact with different solutions for one and 5 minutes. Standard: standard culture method without previous processing of specimens; HOCl: hypochlorous acid; NS: normal saline; PVPI: povidone-iodine.

Fifty CRS patients were enrolled in the first part of the study, including 23 females and 27 males. The mean age was 56.3 years with a range from 25 to 89 years. Among the 50 CRS patients, bacteria were cultured from 27 (54%) patients whose nasal specimens were placed directly into Thanswab tubes. When the sticks were first placed in HOCl solution for one minute, bacteria were cultured from 26 (52%) patients. When the sticks were first placed in NS for one minute, bacteria were cultured from 27 (54%) patients. When the sticks were first placed in alcohol for one minute, bacteria were cultured from 13 (26%) patients. When the sticks were first placed in PVPI solution for one minute, bacteria were cultured from 25 (50%) patients. The bacterial culture rate was significantly lower when the sticks were first placed in alcohol (*p* < 0.001), but the culture rate was not significantly different among the other 4 groups (Figure 1).  Table 1 shows the bacteriologies of all 5 groups. The bacteriological characteristics were similar, with the exception of the alcohol group.

### 3.2. Culture Rates after Management for 5 Minutes

Thirty-two CRS patients were enrolled in the second part of the study, including 13 females and 19 males. The mean age was 52.5 years with a range from 23 to 79 years. Among the 32 CRS patients, bacteria cultured from 14 (43.8%) patients whose nasal specimens were placed directly into Thanswab tubes. When the sticks were first placed in HOCl solution for five minutes, bacteria were cultured from 14 (43.8%) patients. When the sticks were first placed in NS for five minutes, bacteria were cultured from 13 (40.6%) patients. When the sticks were first placed in alcohol for five minutes, bacteria were cultured from 3 (9.4%) patients. When the sticks were first placed in PVPI solution for five minutes, bacteria were cultured from 11 (34.4%) patients. The bacterial culture rate was significantly lower when the sticks were first placed in alcohol (*p* < 0.001), but the culture rate was not significantly different among the other 4 groups (Figure 1).  Table 2 shows the bacteriologies of all 5 groups. The bacteriological characteristics of specimens first placed in HOCl solution were very similar to those processed in the standard manner or first placed in NS.

### 3.3. Comparison of Culture Rates after Management for One Minute and 5 Minutes

When the culture rates of specimens which had been first placed in the different solutions for one minute were compared with those of specimens that had been first placed in the solutions for 5 minutes, the culture rate was lower when sticks were first placed in alcohol for 5 minutes than when sticks were first placed in alcohol for one minute, although this difference was nonsignificant (*p* = 0.088). The culture rates were not significantly different among the other groups based on the duration of processing, that is, 1 versus 5 min.

## 4. Discussion

It has been reported that a low concentration of HOCL solution demonstrated strong antibacterial and antifungal effects [1]. Cho et al. further found that nasal irrigation with a low concentration of HOCL solution improved outcomes of pediatric CRS patients [3]. Patients received nasal irrigation with 30 ml of HOCL for 10 seconds twice a day while sitting or standing. Patients' symptoms improved after HOCl irrigation, but their overall improvement was not greater than that achieved by those who received NS irrigation. However, there was a greater improvement in X-ray scores in patients who received HOCl than in those who received NS irrigation. Nonetheless, the antibacterial effect of HOCl was not evaluated in the study because bacterial culture was not performed.

In our previous study, we evaluated the antibacterial activity of electrolyzed acid water (EAW) on nasal discharge from CRS patients as compared with that of distilled water and alcohol [18]. The active factors responsible for the bactericidal effect of EAW are chlorine-related substances, such as chlorine, hypochlorous acid, and hypochlorous ion. Its antibiotic activity can reach as high as 50 times that of HOCL and kills germs in a short time [19]. Our previous results showed EAW could effectively inhibit the growth of bacteria isolated from nasal secretion, and its antibacterial activity was as effective as alcohol. However, the method of processing the specimens was different from that used in this study. In the aforementioned study, the cotton-tipped sticks were stirred in the glass tube solution in order to dissolve the nasal discharge in the solution. Then, the glass tubes were sent to the clinical microbiology laboratory, and a few drops were taken from glass tubes and applied on the plates in order to develop aerobic and anaerobic cultures. Therefore, the nasal discharge was usually in contact with the solution for more than 30 minutes before inoculation.

In this study, alcohol showed good antibacterial activity as in our previous study [18]. The culture rate for alcohol was 26% when in contact for one minute, 9.4% when in contact for 5 minutes, and 2% in our previous study. The antibacterial effect of alcohol was stronger when it was in contact with nasal discharge for 5 minutes than when it was in contact with nasal discharge for only one minute, although this difference was nonsignificant.

The HOCl solution used in this study was NeutroPhase (NovaBay Pharmaceuticals, Inc., CA, USA) which was stored in a glass bottle. NeutroPhase contains pure HOCl (0.01% concentration in a 0.9% saline solution at pH 3.5–6.5). It is commercially available and easy to use as compared with the production of HOCl by electrolysis of isotonic NS using a device, as performed in other studies [3]. It has been shown that NeutroPhase has rapid bactericidal activity [20]. However, our results found that it did not exert a good antibacterial effect after soaking specimens for one or 5 minutes. PVPI solution is another potent antimicrobial solution, which has been used as a mouthwash [4]. Similarly, it showed no antibactericidal effects after soaking specimens for one and for 5 minutes. Therefore, if antibacterial fluid is used to irrigate the nose to kill germs, the irrigant should stay in the nasal cavities for a duration that is long enough to allow its antibacterial effect to manifest.

## 5. Conclusions

This study showed that HOCl solution did not exert an increased antibacterial effect on bacteria in the nasal discharge of CRS patients within a short time. We postulate that irrigation of the nose with an antibacterial fluid such as HOCl or PVPI for a longer period may be needed to observe any antibacterial activity. Thus, further studies need to be conducted to determine whether soaking nasal discharge in HOCl solution for a duration of longer than 5 minutes would exert a greater antibacterial effect.

## Figures and Tables

**Table 1:** Bacteriology after contact with solution for one minute.

Group^*∗*^	Standard	HOCl	NS	Alcohol	PVPI
Species	No. of Isolates				
Aerobic and facultative bacteria					
Gram-positive					
Coagulase-negative staphylococci	1	2	1		1
Staphylococcus aureus	5	5	5	2	5
Streptococcus pneumoniae	1	1	1	1	1
*Moraxella catarrhalis*	1	1	1		1
Group G *Streptococcus*	1	1	1		1
Gram-negative					
*Haemophilus influenzae*	2	2	2	2	2
*Klebsiella pneumoniae*	4	4	4	2	3
*Klebsiella oxytoca*	1		1		1
*Pseudomonas aeruginosa*	8	6	7	2	6
*Pseudomonas putida*	1	1			
*Enterobacter aerogenes*	1	1	1		1
*Citrobacter koseri*	3	2	3		3
*Escherichia coli*	1	1	1		1
*Stenotrophomonas maltophilia*	2	2	2		2
Total aerobic and facultative bacteria	32	29	30	9	28
Anaerobic bacteria					
Gram-positive					
*Propionibacterium acnes*	1		1	1	2
*Peptostreptococcus micra*	8	7	7	5	8
*Peptostreptococcus anaerobius*	2	2	2	1	2
*Clostridium hastiforme*	1	1	1		1
Gram-negative					
*Fusobacterium nucleatum*	3	3	3	2	3
Total anaerobic bacteria	15	13	14	9	16
Total bacterial isolates	47	42	44	18	44

^*∗*^Standard: the stick was placed directly into a Thanswab tube; HOCl: the stick was placed into a glass tube containing hypochlorous acid solution; NS: normal saline; PVPI: povidone-iodine solution.

**Table 2:** Bacteriology after contact with solution for five minutes.

Group^*∗*^	Standard	HOCl	NS	Alcohol	PVPI
Species	Number of isolates				
Aerobic and facultative bacteria					
Gram-positive					
Coagulase-negative staphylococci		1			
*Staphylococcus aureus*	7	8	6		3
*Staphylococcus* non-*aureus*	1				
*Streptococcus pneumoniae*		1		1	1
Gram-negative					
*Haemophilus influenzae*	2	2	3		2
*Pseudomonas aeruginosa*	2	2	2		2
*Pseudomonas stutzeri*			1		
*Citobacter koseri*	2				
*Escherichia coli*	1		1		
Total aerobic and facultative bacteria	15	15	12	1	8
Anaerobic bacteria					
Gram-positive					
*Propionibacterium acnes*	1	1	1		1
*Peptostreptococcus micra*					2
*Peptostreptococcus magnus*	1	1	1	1	1
Gram-negative					
*Fusobacterium nucleatum*					2
Anaerobic Gram-negative bacillus					1
Total anaerobic bacteria	2	2	2	1	7
Total bacterial isolates	17	17	14	2	15

^*∗*^Standard: the stick was placed directly into a Thanswab tube; HOCl: the stick was placed into a glass tube containing hypochlorous acid solution; NS: normal saline; PVPI: povidone-iodine solution.
